# Differential change in cortical and hippocampal monoamines, and behavioral patterns in streptozotocin-induced type 1 diabetic rats

**DOI:** 10.22038/IJBMS.2018.29810.7197

**Published:** 2018-10

**Authors:** Li-Wei Lin, Fan-Shiu Tsai, Wen-Ta Yang, Shang-Chih Lai, Chun-Chuan Shih, Sheng-Chi Lee, Chi-Rei Wu

**Affiliations:** 1 School of Chinese Medicines for Post-Baccal aureate, I-Shou University, Kaohsiung 82445, Taiwan; 2 Taichung Hospital, Ministry of Health and Welfare, Taichung 402, Taiwan; 3 School of Post-Baccalaureate Chinese Medicine, Tzu Chi University, Hualien 97071, Taiwan; 4 Pintung Branch, Kaohsiung Veterans General Hospital, Pintung 91245, Taiwan; 5 Department of Chinese Pharmaceutical Sciences and Chinese Medicine Resources, College of Pharmacy, China Medical University, Taichung 402, Taiwan

**Keywords:** Biogenic amines, Cerebral cortex, Hippocampus, Memory and learning tests, Mice, Oral glucose tolerance test Type 1 diabetes mellitus

## Abstract

**Objective(s):**

Diabetes mellitus (DM) is a widespread metabolic disorder worldwide. Clinical physicians have found diabetic patients have mild to middle cognitive dysfunction and an alteration of brain monoaminergic function. This study explored the change in various patterns of behavioral models and brain monoamine function under streptozotocin (STZ)-induced type 1 diabetes.

**Materials and Methods:**

We established a type 1 DM model via intravenous injection with STZ (65 mg/kg) in rats. Three weeks after the STZ injection, various behavioral measurements including the inhibitory avoidance test, active avoidance test and Morris water maze were conducted. Finally, all rats were dissected and the concentrations of monoamines and their metabolites in cortex and hippocampus were measured by high performance liquid chromatography with electrochemical detection.

**Results:**

We found that STZ induced type 1 diabetes (hyperglycemia and lack of insulin) in rats. STZ-induced diabetic rats had cognitive impairment in acquisition sessions and long-term retention of the active avoidance test. STZ-induced diabetic rats also had cognitive impairment in spatial learning, reference and working memory of the Morris water maze. STZ significantly reduced concentrations of norepinephrine (NE) in the cortex and dopamine (DA) in the hippocampus, but increased concentrations of DA and serotonin (5-HT) in the cortex 35 days after injection. The concentration of 5-HT in the hippocampus was also significantly increased.

**Conclusion:**

The data suggested that this cognitive impairment after a short-term period of STZ injection might be related to cortical NE dysfunction, differential alteration of cortical and hippocampal DA function, and brain 5-HT hyperfunction.

## Introduction

Diabetes mellitus (DM), a common chronic and progressive metabolic disorder, is characterized by elevated levels of blood glucose (hyperglycemia) and affected 422 million adults in 2014 according to the epidemiological statistics of the WHO. The prevalence of DM in adults was 4.7% in 1980 and increased by up to 8.5% in 2014. The hyperglycemia was resulted from either deficient levels of insulin (type 1 DM) or from defective insulin action (type 2 DM). Hyperglycemia can trigger diabetic ketoacidosis, causing a life-threatening impact in acute complications. Over time hyperglycemia may damage the heart, blood vessels, eyes, kidneys and nerves. Thus, DM can lead to many chronic complications including heart attack and stroke (cardiopathology), kidney failure (renopathology), leg amputation (peripheral microvascular pathology and neuropathology), vision loss (retinopathology), and nerve damage (cerebral neuropathology) (1). Many reports have indicated that DM is associated with decrements in cognitive function and changes in brain structure. Clinical physicians have indicated that DM patients had been shown to have mild to moderate reductions in cognitive function as measured by neuropsychological testing when compared with non-diabetic controls (2). Postmortem research has found that DM patients showed modest cerebral atrophy, increased occurrence of subcortical and brain stem lesions and structural alterations in white matter such as axonal degeneration (3-6). These lesions caused the increased latencies of auditory, visual and somatosensory evoked potentials (6-8). These impairment of sense evoked potentials in diabetic patients caused verbal learning deficits, cognitive impairment or dementia (7, 8). Due to change in brain volume and cerebral microstructure, the progressive alterations of central neurotransmission usually were obviously occurred in DM patients. The activation of central neurotransmission such as acetylcholine, glutamate, and biogenic amines including norepinephrine (NE), dopamine (DA) and serotonin (5-HT) might be related to learning generation and memory consolidation (9). Manjarrez *et al.* indicated that DM patients had lower serotonergic neurotransmission in the auditory cortex which caused lower auditory-evoked potentials (10). There is evidence that DM causes the altered central dopaminergic neurotransmission, especially reflected in changes in DA receptor density and DA transporter activities. These alterations might then lead to diabetic complications such as diabetic chorea and neurocognitive impairment (11, 12). However, there is little literature regarding the change in central noradrenergic neurotransmission in DM patients. Clinical physicians found that diminished cardiac and peripheral NE levels in DM patients can be related to diabetic complications such as cardiopathology and peripheral neuropathology (13, 14).

Animal models for DM range from animals with a spontaneously or hereditary development of diabetes to the chemical ablation of pancreatic β cells or chemical-induced insulin resistance. Some chemical toxins such as streptozotocin (STZ) and alloxan often induce hyperglycemia in rodents (15, 16). STZ, an antibiotic isolated from *Streptomyces achromogenes,* produces pancreatic islet β-cell destruction and is widely used to produce the type 1 DM model in rodents (15, 16). Some researchers have found that rodents intravenously or intraperitonealy treated with STZ presented diabetic syndromes such as polyuria and polydipsia, and exhibited the cognitive impairment in some behavioral tasks including the inhibitory avoidance test, active avoidance test, novel object recognition test, Y maze, and Morris water maze (17-23). However, some researchers have also indicated that STZ-induced diabetic rats had a more prolonged latent period and an increase in the rate of learning of new skills when compared with healthy rats (23). On the other hand, STZ injection caused differential alterations of biogenic amines in various brain areas such as the cerebral cortex, amygdala, cerebellum, hypothalamus, and striatum in rats (24-27). Myhrer pointed out that cerebral acetylcholinergic and monoaminergic activities strongly influenced learning and memory in four behavioral tasks (28). Therefore, the present study investigated the effect of STZ on cognitive function including the inhibitory avoidance test, active avoidance test, and Morris water maze after short-term period of STZ injection in rats. We also measured the concentrations of cortical and hippocampal biogenic amines and their metabolites through high-performance liquid chromatography (HPLC) and electrochemical detection (ECD) in STZ-induced early-stage diabetic rats.

## Materials and Methods

***Drugs***

Streptozotocin (STZ, Sigma-Aldrich Co) was dissolved with 0.1 M citrate buffer (pH 4.5). All standards including 4-hydro-3-methoxymandelic acid (VMA), norepinephrine (NE), 3,4-dihyroxyphenethylamine hydrochloride (DA), 3,4-dihydroxyphenylacetic acid (DOPAC), 5-hydroxytyramine creatine sulfate (5-HT) and 5-hydroxyindoleacetic acid (5-HIAA) were purchased from Sigma-Aldrich Co (USA). Glucose, triethylamine and ortho-phosphoric acid were purchased from Merck (Germany). Sodium 1-octanesulfonate was obtained from Tokyo Kasei Kogyo Co Ltd. (Japan). Ethylenediaminetetraacetic acid disodium salt 2-hydrate (EDTA) and acetonitrile were obtained from JT Baker (USA).

***Experimental animals***

Male Sprague-Dawley rats, weighing 180-230 g, were obtained from the National Laboratory Animal Breeding and Research Center. They were housed in groups of four, chosen at random, in wire-mesh cages (39 cm ×26 cm×21 cm) in a temperature (23 ± 1 ^°^C) and humidity (60%) regulated environment with a 12 hr–12 hr light/dark cycle (light phase: 08:00 to 20:00). The Institutional Animal Care and Use Committee of I-Shou University approved the experimental protocol (IACUC-ISU-9905), and all rats were cared for according to the Guiding Principles for the Care and Use of Laboratory Animals. After one week of acclimatization, the rats were used in the experiments described below.

**Table 1:** Pain thresholds to electric foot-shock 22 days after streptozotocin (STZ, 65 mg/kg, IV) injection in rats

Group	Threshold (mA)	
	Flinch	Jump / Vocalization
VEH	0.76±0.01	0.87±0.02
STZ	0.73±0.03	0.83±0.02

Values are expressed as mean ± SEM. (n = 6 in each group).

**Figure 1:**
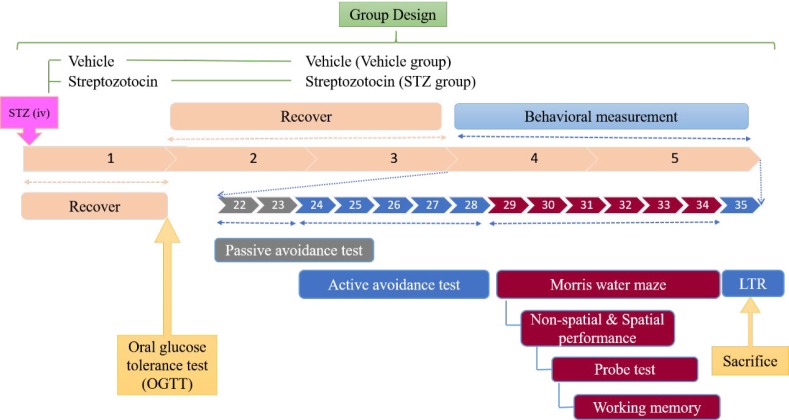
Experimental design of streptozotocin (STZ)-induced type 1 diabetes

**Figure 2:**
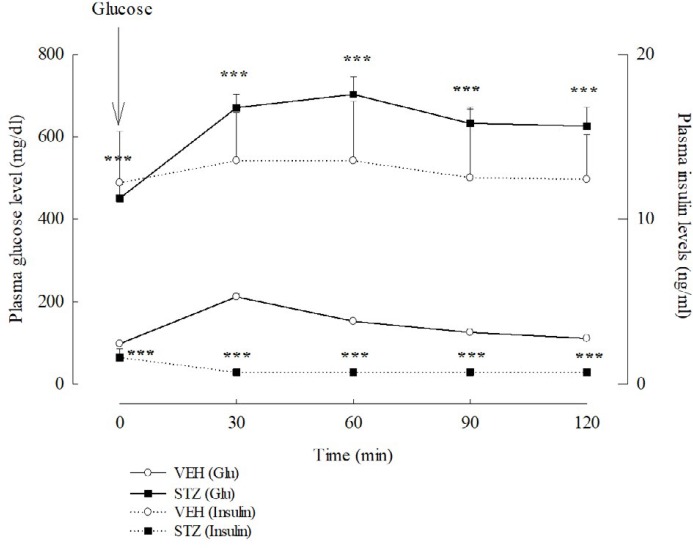
Plasma insulin and glucose (Glu) levels of oral glucose tolerance test (OGTT) 7 days after streptozotocin (STZ, 65 mg/kg, IV) in rats. Values are expressed as mean±SEM (n=6 in each group). *** *P<*0.001 compared to VEH group

**Figure 3:**
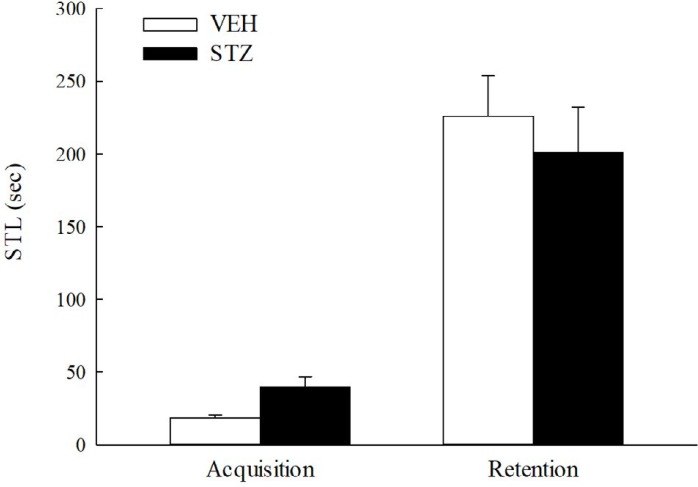
Inhibitory avoidance response from 22 to 23 days after streptozotocin (STZ, 65 mg/kg, IV) injection in rats. Values are expressed as mean±SEM (n = 6 in each group)

**Figure 4:**
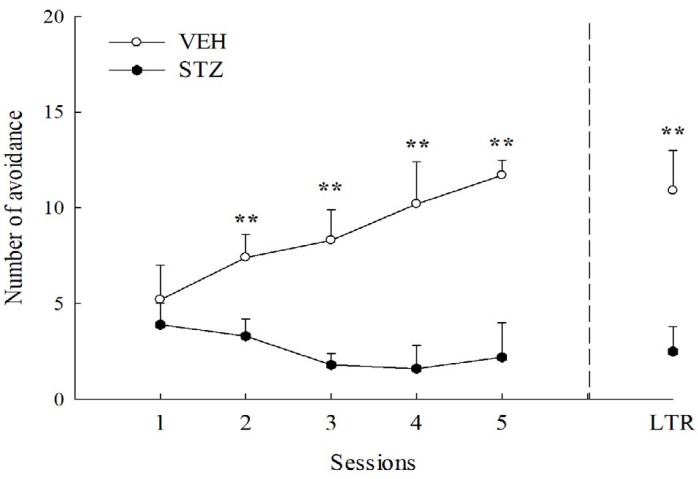
Avoidance response in acquisition sessions and long-term retention (LTR) of active avoidance test in streptozotocin (STZ, 65 mg/kg, IV)-diabetic and vehicle (VEH)-injected rats. Values are expressed as mean±SEM (n = 6 in each group). ** *P<*0.01 compared to VEH group

**Figure 5:**
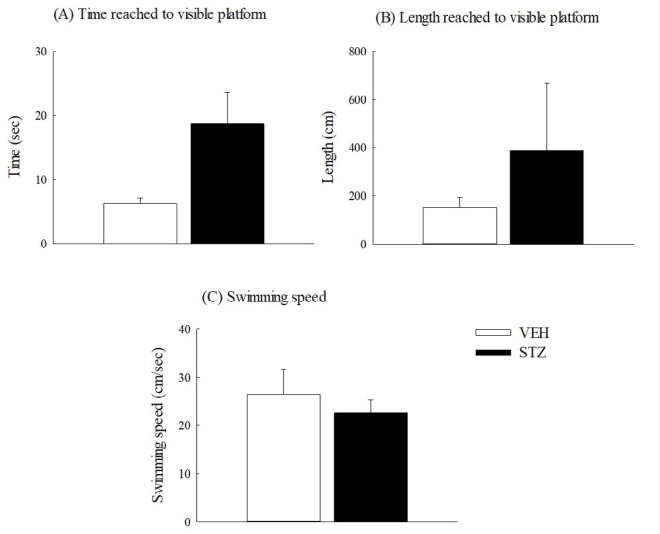
Non-spatial performance of Morris water maze 29 days after streptozotocin (STZ, 65 mg/kg, IV) injection in rats. (A) Time reached to visible platform, (B) length reached to visible platform, (C) swimming speed. Values are expressed as mean±SEM (n=6 in each group)

**Figure 6:**
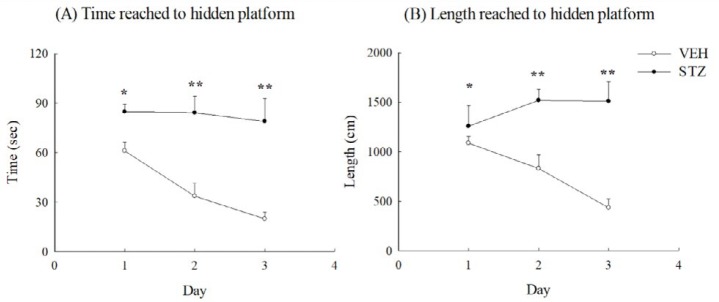
Spatial performance of Morris water maze from 30 to 32 days after streptozotocin (STZ, 65 mg/kg, IV) injection in rats. (A) Time reached to hidden platform, (B) length reached to hidden platform. Values are expressed as mean±SEM (n=6 in each group). * *P<*0.05, ** *P<*0.01 compared to VEH group

**Figure 7:**
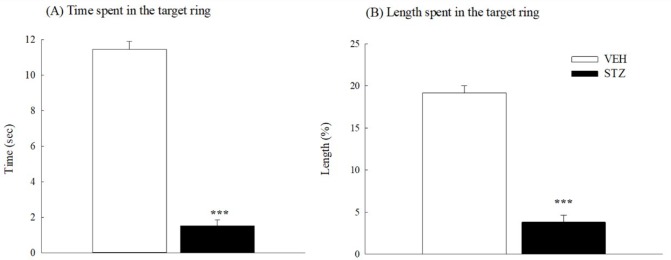
The probe test of Morris water maze 33 days after streptozotocin (STZ, 65 mg/kg, IV) injection in rats. (A) Time spent in the target ring, (B) length spent in the target ring. Values are expressed as mean±SEM (n=6 in each group). *** *P<*0.001 compared to VEH group

**Figure 8:**
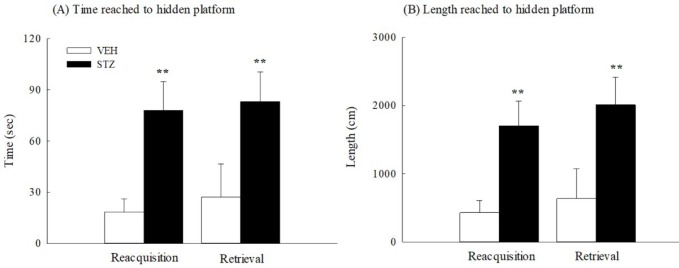
The working memory of Morris water maze 34 days after streptozotocin (STZ, 65 mg/kg, IV) injection in rats. (A) Time reached to hidden platform, (B) length reached to hidden platform. Values are expressed as mean±SEM (n=6 in each group). ** *P<*0.01 compared to VEH group

**Figure 9:**
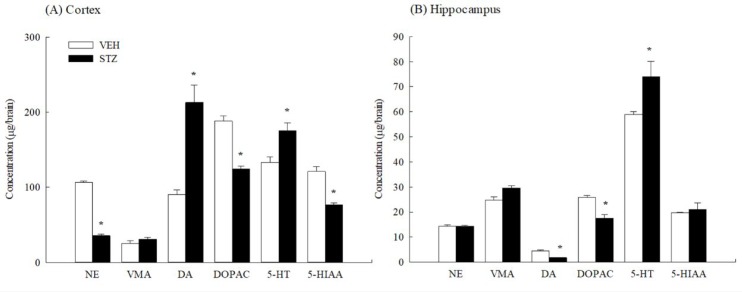
The concentrations of monoamines and their metabolites in (A) cortex and (B) hippocampus of rats 35 days after streptozotocin (STZ, 65 mg/kg, IV) injection. Values are expressed as mean±SEM (n=6 in each group). * *P<*0.05 compared to VEH group

***Experimental design***

The rats of the type 1 DM group (STZ group) were induced by a single intravenous injection of STZ with a dose of 65 mg/kg body weight after overnight fasting (29). The rats of the control group (VEH group) were injected with a vehicle. One week after STZ injection, all rats were held in the stainless holder, blood was collected from the tail, and blood glucose levels were measured using a glucose analyzer (YSI Model 23A, USA). Only rats with blood glucose levels > 200 mg/dl and symptoms of polyuria and polydipsia were considered diabetic and selected for this study. The next day, an oral glucose tolerance test (OGTT) was performed. Three weeks after the STZ injection, behavioral measurements including the inhibitory avoidance test, active avoidance test and Morris water maze were performed. On the following day after the working memory of the Morris water maze (35^th^ day after STZ injection), long-term retention (LTR) of the active avoidance test was evaluated and then all rats were dissected. All behavioral tests were conducted between 09:00 and 17:00. The experimental schedule is shown in Figure 1.

***OGTT***

According to the experimental schedule, the glucose and insulin responses of all rats following oral loading with glucose were evaluated. Blood was collected from the tail vein before and 30, 60, 90, and 120 min after oral administration with glucose (2 g/kg, PO), and were separated into two parts. One part was used to measure the blood glucose level. The other part was drawn into EDTA-coated Eppendorf tubes, and centrifuged at 3,000 rpm for 10 min (4 ^°^C). Plasma was stored at -80 ^°^C and the plasma insulin concentration was determined using a fluorescence radioimmunoassay (FRIA) kit from Abbot (USA).

***Pain threshold to electric stimulation***

The threshold of flinch, jump, or vocalization produced by electric shock was measured by using an inhibitory avoidance apparatus. Each rat was placed in the dark compartment of the inhibitory avoidance apparatus and the shock intensity was manually raised stepwise from 0.5 to 1.0 mA in increments of 0.1 mA until either a flinch, jump or vocalization was observed. The duration of shock was 2 sec and the inter-shock interval was 15 sec (30). The point at which the rat exhibited these responses was recorded.

***Inhibitory avoidance test***

The step-through inhibitory avoidance apparatus consisted of two compartments with a steel-rod grid floor (36 parallel steel rods, 0.3 cm in diameter set 1.5 cm apart). One of the compartments (48×20×30 cm) was equipped with a 20 W lamp located centrally at a height of 30 cm, and the other was a dark compartment of the same size, connected through a guillotine door (5 ×5 cm).

During the acquisition trial (22^nd^ day after STZ injection), each rat was placed in the light compartment with its back to the guillotine door and then the door was opened. The step-through latency (STL) taken by the rat to enter the dark compartment was measured. Once the rat entered the dark compartment, the door was closed. An inescapable scrambled footshock (0.8 mA for 2 sec) was delivered through the grid floor. The rat was removed from the dark compartment 5 sec after footshock. Then the rat was put back into its home cage until the retention trial, which was carried out 24 hr later. The rat was placed in the light compartment again and as in the case of the training trial, the guillotine door was opened and the STL was recorded and used as a measure of retention. An upper cut-off time of 300 sec was set (30).

***Active avoidance test***

Each rat was trained for a two-way active avoidance task in an automated shuttle-box (Ugo Basile, Comerio, Varese, Italy). The apparatus consisted of a shuttle box with two compartments (48×20×22 cm) with a lamp (4 W) and 1000-Hz 75-dB tone, which was used as the conditioned stimulus (CS) and presented for 10 sec until the rat crossed to the opposite compartment. If the rat did not cross to the opposite compartment, it would receive a scrambled 0.8-mA electric foot-shock through the grid floor for 5 sec (unconditional stimulus, US). When the rat crossed to the opposite compartment during the CS, an avoidance response was recorded. The training session consisted of 20 training trials for five consecutive days with inter-trial interval of 30 sec. The long-term memory retention (LTR) was tested one week later, also using 20 trials (31).

***Non-spatial performance of Morris water maze***

To assess visual acuity, spatial performance learning and memory function, the rats were tested in a Morris water maze. The Morris water maze was performed in a circular pool (165 cm in diameter; 60 cm in height) that was made of stainless-steel and filled with water (maintained at 23.0±1.0 ^°^C) to a depth of 35 cm. The swimming performance activity of each rat including the latency to find the platform, total distance traveled, time, and distance traveled in each quadrant was monitored using a CCTV camera and analyzed by a video tracking system (VIDEOMEX-V water maze program, Columbus Instruments, Columbus, USA).

On 29^th^ day after STZ injection, a visible circular platform (10 cm in diameter) was placed into the northeast quadrant of the pool at 1 cm above the water surface. Each rat was given four trials to locate the platform (32). The latency to find the platform was measured as a measure of visual acuity.

***Spatial performance of Morris water maze***

From the 30^th^ to 32^nd^ day after STZ injection, each rat was given four opportunities daily for three consecutive days to find the hidden platform, which was also placed in the center of the northeast quadrant but submerged 1.0 cm below the water surface. Each trial was initiated by placing the rat in the water and facing the pool wall in one of the four quadrants. The daily order of entry into the individual quadrants was randomized so that all four quadrants were used. Each trial was terminated as soon as the rat climbed onto the hidden platform or when 120 sec had elapsed. A rat could stay on the platform for 30 sec. Rats that did not find the platform within 120 sec were put on the platform by the experimenter and were also allowed to stay there for 30 sec. Then, the rat was taken from the platform and the next trial was started after 30 sec. After completion of the fourth trial, each rat was kept warm for an hour and returned to its home cage (33).

***Reference memory of Morris water maze***

The next day following the spatial performance of the Morris water maze (33^rd^ day after STZ injection), the probe test was performed (33). The platform was removed from the pool. The time and distance traveled in the target quadrant were measured during 60 sec.

***Working memory of Morris water maze***

The next day following the probe test of the Morris water maze performance (34^th^ day after STZ injection), the platform was again submerged 1.0 cm below the water surface, but introduced to the pool in the quadrant diametrically opposite its original position. Each rat was given one trial to acclimatize to find the new set of conditions. After four hours, a second trial was carried out in the same manner, but each rat was placed in another quadrant, and the time and length traveled to find the hidden platform were measured (34).

***Measurement of cortical and hippocampal monoamines levels***

After the LTR of the active avoidance test, all rats were decapitated, and their brains rapidly removed from the skull. Following the method by Glowinski and Iversen (35), rat brains were immediately separated into the cortex and hippocampus on ice. All tissues were homogenized with cold 25 mM sodium phosphate buffer (pH=7.4) and centrifuged at 14,000 rpm for 30 min at 4 ^°^C. Then, the supernatants were collected, placed into 0.22 mM Ultrafree MC centrifugal filter units (Millipore, USA), and centrifuged again at 14,000 rpm for 10 min at 4 ^°^C. The collected samples were stored at -80 ^°^C and the concentrations of the cortical and hippocampal biogenic amines (NE, DA, 5-HT) and their metabolites (VMA, HVA, 5-HIAA) were measured using HPLC with ECD. An HPLC Model PM80 (Bioanalytic system Inc), a Data Model M746 (Waters Associates), an electrochemical detector Model LC-4C (Bioanalytic system Inc) and a Bioanalytic system MF-6026 column were used.

***Statistical analyses***

All data are presented as mean±SEM based on six rats in each group. All data including the inhibitory avoidance test, active avoidance test, Morris water maze, plasma glucose and insulin levels as well as the concentrations of cortical and hippocampal biogenic amines were analyzed using the unpaired Student’s t-test. The threshold for statistical significance was *P*<0.05 in all statistical evaluations.

## Results

***OGTT***

One week after VEH or STZ injection, the glucose levels of the VEH and STZ groups were different. The glucose level of the VEH group was 98.14±2.16 mg/dl, and that of STZ group was 450.75±32.73 mg/dl. The average body weight of the VEH and STZ groups were also different three weeks after injection. The average body weight of the VEH group was 325.00±6.45 g (207.50± 4.79 g before injection), and that of the STZ group was 203.06±12.20 g (212.22±4.49 g before injection). The insulin level of the STZ group was significantly lower than that of the VEH group. Thirty minutes after oral administration with glucose, the plasma glucose and insulin levels of the VEH group were elevated when compared with those before treatment. Then, the plasma glucose and insulin levels of the VEH group gradually declined from 60 min to 120 min after glucose treatment (Figure 2). However, the plasma glucose level of the STZ group gradually elevated and reached its highest levels 60 min after glucose treatment. The plasma insulin level of STZ group did not change during the collecting period (Figure 2). There was a significant difference in the tendency and levels of plasma glucose and insulin after glucose treatment between the VEH and STZ groups (*P*<0.001).

***Inhibitory avoidance test and pain threshold to electric stimulation***

Twenty-two and twenty-three days after STZ injection, the STL of the STZ group in the acquisition trial was longer (but not with significant statistical meaning) than that of the VEH group (*P*>0.05). There was no difference between the STL of the VEH or STZ groups in the retention trial (*P*>0.05) (Figure 3). As shown in Table 1, the flinch and vocalization thresholds of the STZ group were not different to those of the VEH group (*P*>0.05).

***Active avoidance test***

The avoidance numbers of the VEH group gradually increased during consecutive 5-day training sessions. However, there was a decrease tendency in the avoidance numbers of the STZ group during consecutive 5-day training sessions (from 24 to 28 days after STZ injection). There was a significant difference in the avoidance numbers during consecutive 5-day training sessions between the VEH and STZ groups (*P*<0.01) (Figure 4). There was also a significant difference in the avoidance response of long-term memory after 7-day retention (35^th^ day after STZ injection) between the VEH and STZ groups (*P*<0.01) (Figure 4).

***Morris water maze***

Twenty-nine days after STZ injection, the STZ group spent a longer time and distance (but not with statistical significance) reaching the visible circular platform than those spent by the VEH group (*P*>0.05) (Figure 5 (A-B)). However, the swimming speed of the STZ group was slower (but not with statistical significance) than that of the VEH group (*P*>0.05) (Figure 5 (C)).

For the next consecutive three days, the VEH group took a gradually declining time and distance to reach the hidden platform on the spatial performance trials of the Morris water maze. The STZ group spent more time and 

distance to reach the hidden platform and the time did not have the same declining tendency during the 3-day spatial performance trials (*P*<0.05, *P*<0.01) (Figure 6 (A-B)).

In the probe test (33 days after STZ injection), the time spent in the target ring and the length ratio (traveled length in the target ring per total traveled length) of the STZ group were significantly and obviously shorter than those of the VEH group (*P*<0.001) (Figure 7(A-B)). Thirty-four days after STZ injection, the STZ group also took a longer time and distance to reach the circular hidden platform than those spent by the VEH group in the acquisition and retrieval trials of working memory (*P*<0.01) (Figure 8 (A-B)).

***Cortical and hippocampal monoamine levels***

Thirty-five days after STZ injection, the STZ reduced cortical NE, DOPAC and 5-HIAA levels when compared with the VEH group. However, cortical DA and 5-HT levels were increased after STZ injection (*P*<0.05) (Figure 9(A)). STZ also increased the hippocampal 5-HT level when compared with the VEH group but decreased hippocampal DA and DOPAC levels (*P*<0.05) (Figure 9(B)).

## Discussion

Clinical physicians have indicated that DM patients had mild to moderate cognitive dysfunction compared with healthy controls (2). STZ is widely used to produce the type 1 DM model in rodents and induced cognitive impairment in some behavioral tasks including the inhibitory avoidance test, active avoidance test, novel object recognition test, Y maze and Morris water maze (17-23). The current study was conducted with various behavioral tasks including the inhibitory avoidance test, active avoidance test and Morris water maze to investigate the cognitive function in the STZ-induced diabetic rats. First, we established a type 1 DM model *via* IV injection with STZ (65 mg/kg) in rats. STZ caused hyperglycemia, hypoinsulinemia and some diabetic syndromes such as polyuria and polydipsia. There was a higher, but slower uptrend in the blood glucose levels of STZ-diabetic rats than those of normal rats in the OGTT. This phenomenon might be due to increased insulin secretion after the oral glucose treatment in normal rats, and the lack of insulin secretion in STZ-diabetic rats.

Inhibitory avoidance and active avoidance are aversive learning models that used an electrical footshock (aversive event) to induce memory formation. Due to very different results from previous reports regarding the inhibitory avoidance response of STZ-diabetic rodents (18, 21, 36, 37), we first compared the inhibitory avoidance response of the STZ-diabetic rats with that of normal rats according to the behavioral measurement schedule. Inhibitory avoidance is defined as the suppression of the innate preference for the dark compartment of the test apparatus following exposure to an inescapable shock. Rats must learn to suppress a motor response to avoid exposure to the preferred area associated with the aversive event. This paradigm is simple learning model and is based on emotional aversive learning involving the amygdala. We found that STZ did not impair the inhibitory avoidance response in the retention trial (twenty-three days after induction) in rats. This result was similar to Jing *et al.* (38), but differed to other reports where STZ-diabetic rodents had more or less inhibitory avoidance behavior (18, 21, 36, 37). As the inhibitory avoidance response can be related to sensitivity to nociceptive stimuli or motor effects during training (30), we further observed motor activity during training and the pain threshold for electric footshock in the inhibitory avoidance apparatus. STZ-diabetic rats had longer latency but not with any statistical significance in the acquisition trial than the normal rats. There was no difference in pain threshold between the STZ-diabetic rats and normal rats, although chronic hyperglycemia has caused peripheral neuropathy and hyperalgesia in animal studies and clinical reports (39-41). Therefore, we suggested that STZ did not cause peripheral neuropathy and aversive learning (punishment) impairment three weeks after injection in rats. One report indicated that increased inhibitory avoidance response in STZ-diabetic rodents could be due to increased sensitivity to shock, and STZ-diabetic and normal rodents had a similar response at a higher shock intensity (37). This difference in the inhibitory avoidance response of STZ-diabetic rodents might be related to the interval between the STZ injection and behavioral measurement (three weeks vs two months), behavioral paradigm (one-trial vs multi-trial), and shock intensity.

Second, we further compared the avoidance response of the acquisition sessions and long-term retention in two-way active avoidance test (positive reinforcement) of STZ-diabetic rats with that of normal rats as previous reports have also shown the different results where STZ could impair or facilitate the active avoidance response of STZ-diabetic rodents (23, 42). Two-way active avoidance is defined as Pavlovian contextual fear instrument learning model. Rats must perform a discrete response of a low probability, *e.g.*, running from one side of a two-compartment box to the other when a conditioned stimulus (CS) is presented to escape or avoid the unconditioned stimulus. This paradigm is a relatively complex learning model and is also based on emotional associative learning involving the amygdala, hippocampus, thalamus, and cortex. The present result was consistent with those of Kucukatay *et al.* (42), who found that STZ impaired the avoidance response of five-day acquisition sessions and long-term retention in a two-way active avoidance test in rats. Therefore, we suggested that STZ impaired the active avoidance response and long-term memory, but not the inhibitory avoidance response three weeks after induction in rodents. This difference of cognitive decline caused by STZ-induced diabetes on aversive behavioral tasks might be related to the complexity and characteristics of behavioral performance (punishment vs associative memory), and the interval between the STZ injection and behavioral measurement (two weeks vs two months).

Finally, many researchers have demonstrated that STZ impairs spatial performance and reference memory in the Morris water maze from one to six months after induction (18, 43). The Morris water maze was designed as a method to assess spatial or place learning. This spatial learning test for rats relies on distal cues to navigate from start locations around the perimeter of an open swimming arena to locate a submerged escape platform. This paradigm is a relatively complex learning model and is based on spatial learning involving the amygdala, hippocampus, and prefrontal cortex. This investigation demonstrated that STZ-diabetic rats exhibited impaired spatial performance in the Morris water maze with a hidden platform. STZ-diabetic rats spent more time in the border zone of the pool, suggesting a reduced comprehension of the maze. Place preference was impaired, possibly reflecting a deficit in spatial learning, and possibly secondary to the impaired understanding of the maze. The spatial learning of rats in a water maze is influenced by swimming speed, endurance and vision (44). As STZ caused the deficits in the visual acuity, attention, and motor abilities of rats, non-spatial performance (visible platform) in the Morris water maze was carried out before the spatial performance (hidden platform). In the visual version of the Morris water maze, STZ-diabetic rats spent more time escaping latency and distance to the visible platform than normal rats. The swimming speed of STZ-diabetic rats was slower than that of normal rats. However, these differences in the visual version between the STZ-diabetic and normal rats showed no statistical significance. This result is consistent with other reports where STZ did not reduce visual function and induce an early-stage of diabetic retinopathology within six weeks after induction (45). Together, the above experiments indicate that the impaired performance of STZ-diabetic rats in the spatial version of the water maze was related to spatial learning impairment and the difficulties in learning the procedures of the maze. The hidden platform did not appear to present a sufficiently strong cue for diabetic rats to switch from a strategy of trying to escape from the edges of the pool to a strategy of locating the centrally located platform. This investigation also found that STZ-diabetic rats exhibited impaired reference and working memory in the Morris water maze. Thus, STZ not only impaired learning acquisition and short-term memory, but also impaired memory retrieval and long-term memory in rats one-month after injection.

Myhrer pointed out that cerebral monoaminergic activities strongly influenced learning and memory in four behavioral tasks (28). Early studies indicated that STZ-diabetic rats exhibited alterations in the contents of particular brain monoamines (24-27). Our present study revealed that STZ significantly reduced concentrations of NE in the cortex and DA in the hippocampus, but increased concentrations of DA and 5-HT in the cortex thirty-five days after injection. The concentration of 5-HT in the hippocampus was also significantly increased. The reduction in cortical NE contents caused by STZ was consistent with most reports where NE contents in most brain regions were almost reduced by STZ (46). NE activity in the cortex, amygdala, and hippocampus alters memory processing and intracisternal injection with NE facilitates memory consolidation (47). The increase in cortical and hippocampal 5-HT contents caused by STZ was consistent with reports where brain 5-HT contents were increased in STZ-diabetic or alloxan-diabetic rats. Cerebral and hippocampal serotonergic systems usually play a modulatory role in memory function. Contextual fear learning and memory was markedly enhanced in central serotonin-deficient mice (48). Therefore, we suggested that the differential cognitive dysfunction caused by STZ might be related to the differential alteration in brain monoaminergic function such as the reduction in cortical NE contents and hippocampal DA contents, and the increase in brain 5-HT contents.

## Conclusion

Taking all the above observations into consideration, intravenous injection with STZ (65 mg/kg) in rats caused cognitive deficits in some behavioral tasks such as the active avoidance test and Morris water maze but not the passive avoidance test. However, STZ did not alter pain threshold, visual acuity, and motor activity within one-month after injection in rats. There were lower cortical NE and hippocampal DA levels in the STZ-diabetic rats than those of the normal rats. However, there were higher cortical and hippocampal 5-HT levels in the STZ-diabetic rats than those of the normal rats. Therefore, the present study suggests that STZ could induce diabetic syndromes and impair the mild cognitive function in rats. The cognitive dysfunction caused by STZ must be dependent on the complexity of behavioral models within one-month after induction and related to the alteration of monoamine neurotransmitter systems, particularly in brain regions such as NE in the cortex, DA in the hippocampus, and 5-HT.
